# The 39-week rule and term stillbirth: beneficence, autonomy, and the ethics of the current restrictions on early-term labor induction in the US

**DOI:** 10.1186/1471-2393-15-S1-A9

**Published:** 2015-04-15

**Authors:** James M Nicholson

**Affiliations:** 1 Department of Family and Community Medicine, Hershey Medical Center, Pennsylvania State University, Hershey, Pennsylvania, USA

## 

The cumulative risk of term stillbirth, i.e., the death of a fetus in utero on or after 37 weeks 0 days of gestation, increases with increasing gestational age throughout the term period (37 weeks 0 days – 41 weeks 6 days)[1]. Despite this fact, a rule – called **the 39-week rule** – was established in 2009 that restricts labor induction in the 37^th^ and 38^th^ week of pregnancy (i.e., in the “early-term period”) unless an accepted/approved “indication” is present (Table 1). [2] The 39-week rule is now a strict clinical guideline that is enforced by professional organizations, governmental agencies and the medical insurance industry[3,4,5]. The 39-week rule means that a pregnant woman who has an identifiable risk factor for stillbirth but who does not have an accepted “indication” for labor induction has no choice but to wait until at least 39 weeks 0 days before she can be delivered. Unfortunately, the strict application of the 39-week rule has probably led to hundreds early-term stillborn infants in the US over the past few years[6,7].

**Table 1:** Accepted Indications for Labor Induction

Late-term pregnancy (> 41 weeks 0 days of gestation)
Severe fetal growth restriction (fetus not growing, < 5%)

Rupture of membranes without labor

Severe pre-eclampsia (hypertension of pregnancy)

Chorio-amnionitis (amniotic fluid infection)

Failed antenatal testing (possible fetal compromise)

Significant oligohydramnios (AFI < 6)

The purpose of this presentation was to disclose major problems with the development, application and ethics of the 39-week rule. Firstly, the evidentiary foundation of the 39-week rule is composed almost entirely of observational studies (i.e., Level 2 evidence) that contain a variety of serious flaws including confounding by indication, [8-10] confounding by situation, [10-12] selection bias, [13] misclassification bias, [14] incorrect modelling, [8-10] and the use of data from pre-37 week deliveries [10,15] and/or pre-labor cesarean deliveries.[16,17]. Secondly, these observational studies report magnitudes of association between early-term non-indicated labor induction and adverse birth outcomes (as measured in relative risk [RR], odds ratio [OR]) that are not large enough to be used to claim the identification of an underlying “truth” (i.e., that early-term non-indicated labor inductions per se *cause* adverse birth outcomes). [18] Thirdly, the evidentiary foundation ignores recent higher-quality research that suggests that early-term non-indicated labor induction might provide significant benefits[19,20,21]. Fourthly, the 39-week rule was created by a process that chose the relatively arbitrary “cut-point” of 39 weeks 0 days of gestation [22], failed to consider the potential importance of intermediate levels of prenatal risk [23], ignored the opinions and experience of non-academic providers [24], and excluded input from the general public. Fifthly, the 39-week rule ignores the primary importance of the medical ethical principle of Autonomy [25,26]. Autonomy represents the concept that a patient, given that she has a reasonably good understanding of risk and benefit, has the right to either request or refuse any given reasonable medical therapy. The 39-week rule prevents a woman from requesting and receiving a non-indicated induction of labor in the early-term period of pregnancy. The reason given for this restriction on patient autonomy in the setting of early-term non-indicated labor induction is the application of another medical ethical principle called Beneficence [2]. Beneficence represents the concept that a provider has the obligation to provide a patient with the best treatment(s) available. However, as noted above, it is unclear if the use of labor induction in the absence of an accepted “indication” in the early-term period of pregnancy provides more harm than benefit. The 39-week rule is not supported by the type of evidentiary foundation that is generally needed to restrict patient Autonomy [27,28].

In summary, the 39-week rule is not supported by high-quality evidence, its strict application unjustifiably obstructs patient autonomy, and it may actually cause harm in the form of early-term stillbirth. Because of these problems the 39-week rule should be modified, made optional, or withdrawn. Patients should be able to request and receive early-term labor induction if they believe that such an intervention is in the best interest of themselves and/or their fetus.
